# Data Adaptive Analysis on Vertical Surface Deformation Derived from Daily ITSG-Grace2018 Model

**DOI:** 10.3390/s20164477

**Published:** 2020-08-11

**Authors:** Weiwei Li

**Affiliations:** 1 College of Geodesy and Geomatics, Shandong University of Science and Technology, Qingdao 266590, China; skd994691@sdust.edu.cn; 2 Key Laboratory of Geomatics and Digital Technology of Shandong Province, Qingdao 266590, China

**Keywords:** data adaptive, enhanced harmonic analysis, ITSG-Grace2018, daily solution, time-varying signal

## Abstract

With the widely used monthly gravity models, it is hard to determine the sub-monthly variations. Thanks to the high temporal resolution, a daily ITSG-Grace2018 gravity model is employed to derive the vertical deformation of the China region in 1.0° × 1.0° grids. The standard deviations of residuals between the daily and monthly averaged displacement range from 1.0 to 3.5 mm, reaching half of the median residuals, which indicates that a higher temporal resolution gravity model is quite necessary for the analysis of crustal displacement. For the signal analysis, traditional least square (LS) is limited in its analysis of signals with constant amplitude. However, geophysical signals in a geodetic time series usually fluctuate over long periods, and missing data happen. In this study, the data adaptive approach called enhanced harmonic analysis (EHA), which is based on an Independent Point (IP) scheme, is introduced to deal with these issues. To demonstrate the time-varying signals, the relative differences between EHA and LS are calculated. It illustrates that the median percentage of epochs at grids with a relative difference larger than 10% is 69.7% and the proportions for the ranges of 30%, 50%, and 70% are about 30.1%, 18.4%, and 13.0%, respectively. The obvious discrepancy suggests the advantage of EHA over LS in obtaining time-varying signals. Moreover, the spatial distribution of the discrepancy also demonstrates the regional characteristics, suggesting that the assumption of constant amplitude is not appropriate in specific regions. To further validate the effectiveness of EHA, the comprehensive analysis on the different noise types, number of IPs, missing data, and simultaneous signals are carried out. Specifically, EHA can deal with series containing white or color noise, although the stochastic model for the color noise should be modified. The signals are slightly different when selecting different numbers of IPs within a range, which could be accepted during analysis. Without interpolation, EHA performs well even with continuously missing data, which is regarded as its feature. Meanwhile, not only a single signal but also simultaneous signals can be effectively identified by EHA.

## 1. Introduction

The satellite gravity mission Gravity Recovery and Climate Experiment (GRACE) observed the time-variable gravity field of the Earth from 2002 to 2017. GRACE data indicate large seasonal variations in gravity, which are related to climate-driven fluxes of surface water [1]. The seasonal redistribution of surface mass deforms the Earth; thus, GRACE is an important tool for monitoring deformation [2]. The updates on monthly gravity models were performed continuously, and the recent monthly gravity model is Release 06 (RL06), which is provided by three institutions (Center for Space Research (CSR), GeoForschungsZentrum (GFZ), and Jet Propulsion Laboratory (JPL)). With the improvement in the processing algorithm and background gravity model [3], RL06 shows its advantage in terrestrial water storage [4,5], polar motion estimation [6], and so on. However, the general temporal resolution of the GRACE gravity model is monthly, which limits obtaining the sub-monthly mass variation. While there are fast mass variation phenomena occurring on time scales with shorter than month, the traditional monthly gravity models cannot detect sub-monthly drought and flood, such as the major flood events in Ganges-Brahmaputra in 2004 and 2007 [7]. Furthermore, to study the contribution of geophysical signals on Global Navigation Satellite System (GNSS) time series, their temporal resolutions should be consistent. Actually, the daily or weekly GNSS coordinate time series are common. Therefore, the general practice is averaging daily or weekly GNSS time series to monthly to keep consistent with the monthly gravity field [8,9,10]. In fact, the reduction of temporal resolution would affect the signal extraction and its accuracy [11]. To increase the temporal resolution, various groups have developed 10-day and weekly interval gravity models. The Institute of Theoretical Geodesy and Satellite Geodesy (ITSG) of the Graz University of Technology incorporates the new RL06 background gravity model with Kalman smoother in providing an ITSG-Grace2018 daily model [12]. Until now, the characteristics of displacement derived from daily ITSG-Grace2018 have not been studied yet.

For monitoring deformation, the combination of geophysical models is another good option in addition to GRACE. The geophysical models satisfy the demand of higher temporal and spatial resolution and many studies have highlighted the feasibility of analyzing the mass loading on GNSS coordinate time series [10,13]. However, the contribution they can explain is generally lower than that which is GRACE-derived. It may due to the area coverage of geophysical models, such as the GLDAS (Global Land Data Assimilation System), which does not cover Antarctica [13] and only has a limited storage capacity [14]. Meanwhile, the consistency among different models, in other words, whether any of the models for atmosphere, ocean, and terrestrial water storage can be freely combined to get the mass variation is still an open question [13,15].

To understand the seasonal variation in displacement time series, many approaches have been employed. The traditional approach is least square (LS) estimator, which is under the assumption of temporally constant amplitude. However, the geophysical causes are unlikely to remain unchanged during a long period. Therefore, its underlying assumption is unrealistic. To accommodate temporal variations of periodic signals, non-parametric approaches such as Empirical Mode Decomposition (EMD) [16] and Singular Spectrum Analysis (SSA) [17,18] are introduced. However, EMD is prone to have the end effect, while the window length and component number are two deterministic factors for SSA. Furthermore, both methods are not efficient when missing data are present [19,20]. A new data adaptive approach, enhanced harmonic analysis (EHA), based on an Independent Point (IP) scheme, is not limited to missing data. It has been successfully applied to analyze the tide variation [21] and determine the temporal changes of the M2 tide in the Gulf of Maine [22]. Since missing data often happens, in this regard, EHA shows its own feature in data analysis.

The rest of this paper is organized as follows: Section 2 introduces the data and EHA methodology. The 1.0° × 1.0° grid displacements throughout China are calculated, and their time-varying characteristics of annual signal extracted with EHA are presented in Section 3. To further confirm the effectiveness of EHA, the impact of different noise, different numbers of IPs, missing data, and simultaneous periodic signals are assessed in Section 4. The characteristics of annual signal and EHA are discussed in Section 5. Finally, the concluding remarks are drawn in Section 6.

## 2. Data Sources and Methodology

### 2.1. Data and Preprocessing

**Figure 1:**
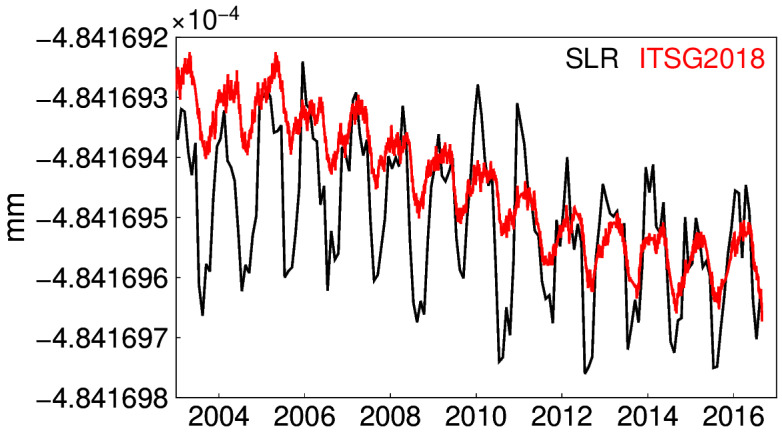
C_20_ from satellite laser ranging (SLR) and Institute of Theoretical Geodesy and Satellite Geodesy Gravity Recovery and Climate Experiment (ITSG-Grace2018).

The ITSG-Grace2018 release is based on Level-1B Release 03 data and the AOD1B (Atmosphere and Ocean De-Aliasing Level-1B) Release 06 de-aliasing product. The daily solutions are Kalman smoothed and constrained within the least square adjustment. Therefore, no spatial filtering is necessary for daily solutions [7,23]. To avoid substantial uncertainties, the early beginning and the end period of GRACE is excluded [24]. Thus, the investigated period here is fixed from January 2003 to August 2016. Meanwhile, the maximum degree of this daily model is 40. Since the intra-annual features of the C_20_ time series of ITSG-Grace2018 are similar to those of the satellite laser ranging (SLR) solution (Figure 1), we do not replace C_20_ in this work.

**Figure 2:**
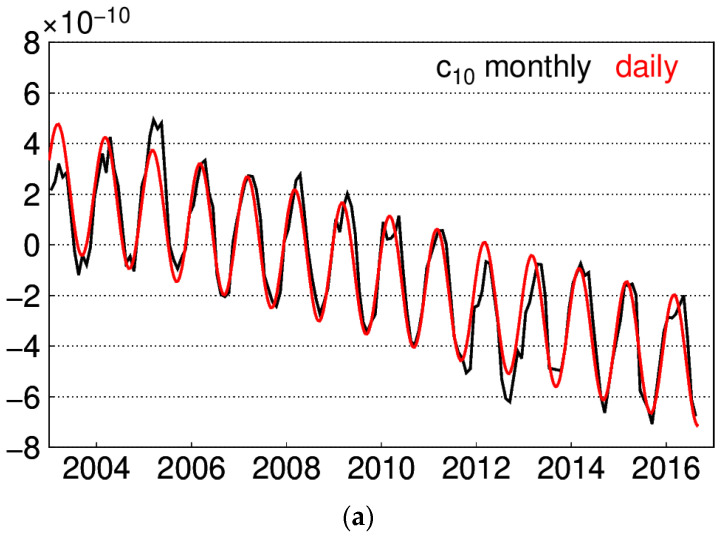

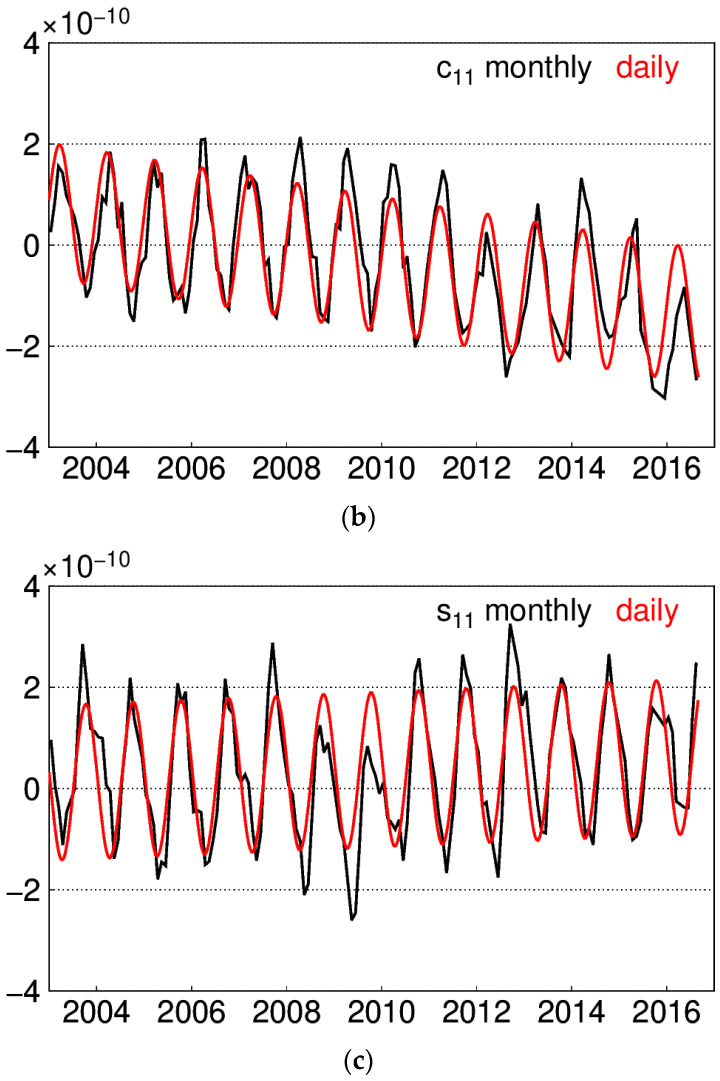
Monthly and daily interpolated degree-1 time series: (**a**) c_10_ series; (**b**) s_10_ series; (**c**) s_11_ series.

Due to the significant periodic characteristics of monthly degree-1 time series from SLR [25], the daily degree-1 coefficients are obtained by harmonic fitting of the monthly resolution. The fitting model can be expressed as:

(1) cs(t)=a+bt+ccosωt+dsinωt+e(t)

where cs represents the degree-1 harmonic coefficients, a,b,c,d are the fitting parameters to estimate, and ω is the angular frequency of the periodic signal. Using the square root of the sum of residuals as penalty starting at the initial value of ω, the iteration is performed to search the minimum value. Thus, ω is determined, and the fitting parameters are calculated through LS. Since the investigated period spans from January 2003 to August 2016, the same period for degree-1 is used to obtain the fitting parameters in Equation (1). The monthly degree-1 adopted is the RL06 from CSR, which is downloaded from [26]. The monthly and interpolated daily degree-1 time series are presented in Figure 2.

Since Kalman smoother was applied in the daily gravity model generation, there are no gaps in the series even without observations. To get reliable results, all days without any or insufficient GRACE data (less than 10,000 observations) were excluded from the analysis [23], with the missing rate of 16.15% during the investigation period. After the preprocessing of harmonic coefficients, the vertical displacement caused by mass variation can be calculated with:

(2) $$u(\theta ,\lambda )\;=\;R\sum_{l=1}^{40}\sum_{m=0}^{l}{\tilde{P}}_{l,m}(\cos\theta )[\Delta C_{lm}\cos(m\lambda )\;+\;\Delta S_{lm}\sin(m\lambda )]\frac{h_{l}^{\prime }}{1+k_{l}^{\prime }}$$

where θ,λ are the colatitude and longitude of the station; R is the earth radius; ${\tilde{P}}_{lm}$ is the normalized Legendre function of degree l and order m; $\Delta C_{lm},\Delta S_{lm}$ are the daily harmonics variation reference to the mean harmonics during our investigated period; and $h_{l}^{\prime }$,$\;k_{l}^{\prime }$ are the love numbers of degree l, which are from the Preliminary Reference Earth model (PREM). In order to get the complete mass variations including atmosphere and ocean, the de-aliasing products are considered.

### 2.2. Data Adaptive Analysis Methodology

With the linear trend and two periodic signals, the traditional constant harmonic fitting is as follows:

(3) $$u(t)=a+bt+\sum_{j=1}^{2}(c_{j}\cos\omega_{j}t+d_{j}\sin\omega_{j}t)+e(t)$$

where b is the linear trend; for j=1, $c_{1}$ and $d_{1}$ are the harmonics for the annual signal ($\omega_{j}=2\pi$); and for j=2, $c_{2}$ and $d_{2}$ are the harmonics for the semi-annual signal ($\omega_{j}=4\pi$). In this case, for annual signal, the amplitude is represented as $\sqrt{c_{1}^{2}+d_{1}^{2}}$, which is unchanged with time. Through LS, the linear and the seasonal signal of constant amplitudes can be obtained. While in fact, the parameters to estimate in Equation (3) are related with time; to be more rigorous, they should be expressed as:

(4) $$u(t)=a+b(t)t+\sum_{j=1}^{2}(c_{j}(t)\cos\omega_{j}t+d_{j}(t)\sin\omega_{j}t)+e(t)$$

How to get the time-varying parameters is the issue that data-adaptive EHA solves. Its main idea is using the harmonic parameters for the selected Independent Points (IPs) to interpolate the remaining epochs. These IPs are usually uniformly distributed, and the numbers are p and q, respectively. Thus, the parameters are as follows:

(5) $$\begin{array}{l} b(t)=\sum_{i=1}^{p}f_{t,i}b_{i}\\ c_{j}(t)=\sum_{i=1}^{q}f_{t,i}c_{i,j}\\ d_{j}(t)=\sum_{i=1}^{q}f_{t,i}d_{i,j} \end{array}$$

where $f_{t,i}$ is the interpolation weight for the ith IP at time t; here, the cubic spline is suggested to adopt [21]. By substituting (5) into (4), we can derive *N* equations in Equation (6) for solving the unknown $b_{i},c_{i,j},d_{i,j}$ by LS. Thus, the time-varying trend and temporal variation of seasonal signal can be quantified.

(6) $$\left\{ \begin{array}{l} u(t_{1})=a+t_{1}\sum_{i=1}^{p}f_{t,i}b_{i}(t_{1})+\sum_{j=1}^{2}\left(\cos\omega_{j}t_{1}\sum_{i=1}^{q}f_{t,i}c_{i,j}+\sin\omega_{j}t_{1}\sum_{i=1}^{q}f_{t,i}d_{i,j}\right)\\ u(t_{2})=a+t_{2}\sum_{i=1}^{p}f_{t,i}b_{i}(t_{2})+\sum_{j=1}^{2}\left(\cos\omega_{j}t_{2}\sum_{i=1}^{q}f_{t,i}c_{i,j}+\sin\omega_{j}t_{2}\sum_{i=1}^{q}f_{t,i}d_{i,j}\right)\\ \vdots \\ u(t_{N})=a+t_{N}\sum_{i=1}^{p}f_{t,i}b_{i}(t_{N})+\sum_{j=1}^{2}\left(\cos\omega_{j}t_{N}\sum_{i=1}^{q}f_{t,i}c_{i,j}+\sin\omega_{j}t_{N}\sum_{i=1}^{q}f_{t,i}d_{i,j}\right) \end{array}\right.$$

It is easy to find that the constant periodic signal is obtained when the number of IPs, q, is equal to 1. Once the number of IPs gets larger than 2, it means that more variations are reflected. However, it does not mean that the more IPs, the better. The avoiding of over-parameterization should be kept in mind. Meanwhile, the number of IPs can be different for different periodic signals. Actually, the determination of number of IPs is a reasonable compromise.

## 3. Results and Analysis

### 3.1. Characteristics of Daily Vertical Displacements

**Figure 3:**
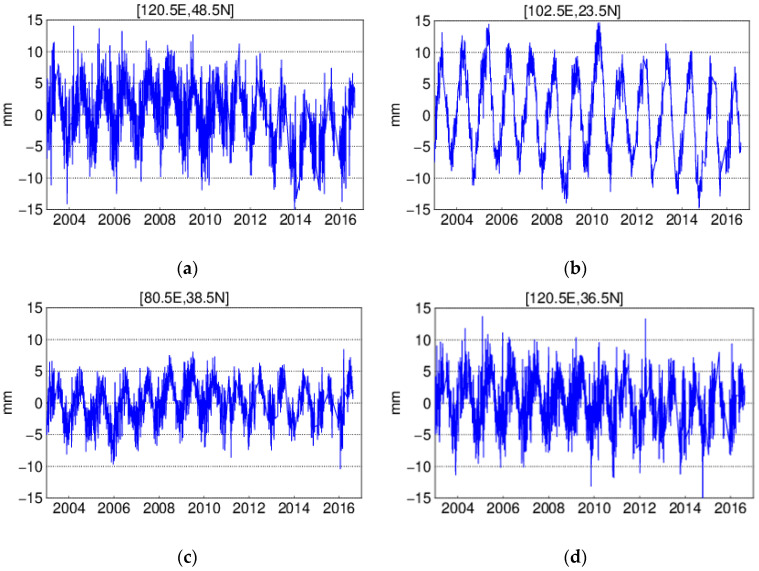
Vertical daily displacement derived from GRACE at four selected grids: (**a**) at grid (120.5 E, 48.5 N); (**b**) at grid (102.5 E, 23.5 N); (**c**) at grid (80.5 E, 38.5 N); (**d**) at grid (120.5 E, 36.5 N).

**Figure 4:**
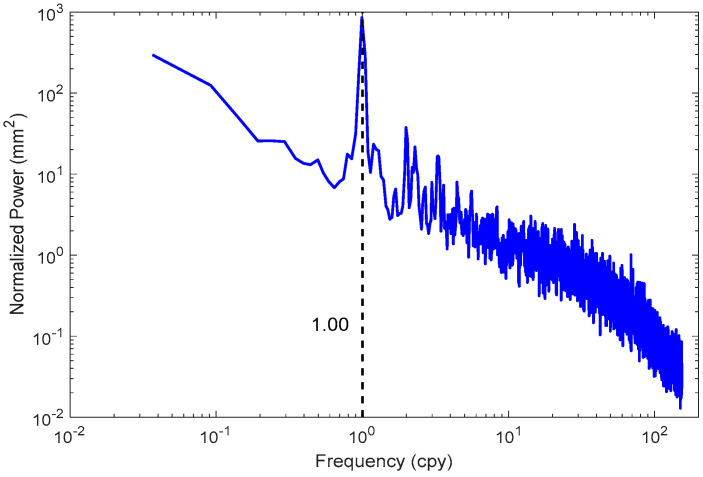
Stacked periodograms of displacement time series of all the grids.

**Figure 5:**
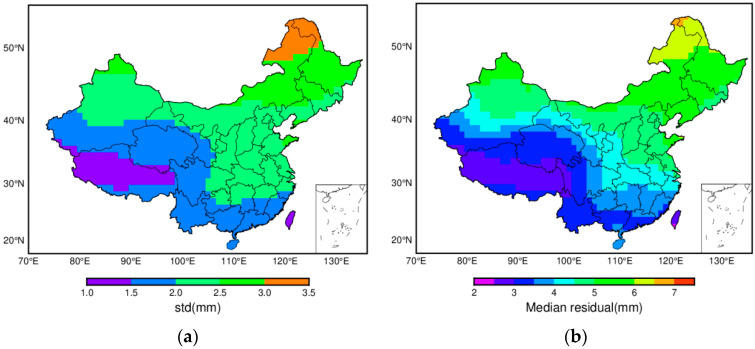
Standard deviations (**a**) and median (**b**) of residuals between daily and the monthly average.

The vertical displacement covering China in 1.0° × 1.0° are calculated according to Equation (2). Here, we take (120.5 E, 48.5 N) grid on the North side, (102.5 E, 23.5 N) on the South side, (80.5 E, 38.5 N) on the West side, and (120.5 E, 36.5 N) on the East side as an example; the time series derived from the ITSG-Grace2018 daily gravity model are shown in Figure 3. They highlight the variation at daily scale and the significant annual signal. The Lomb–Scargle normalized periodogram is employed to detect the periodic characteristics, which confirms the existence of the annual signal (see Figure 4). Although monthly gravity models are common, they cannot distinguish the variation at a smaller temporal scale. To check whether monthly averaged gravity could represent the variation during one month or not, the standard deviations and the median of the residuals between daily displacements and monthly averages are presented in Figure 5. It is apparent that the standard deviations reveal distinct regional features, which increase from southwest to northeast, ranging from 1.0 to 3.5 mm. In terms of the median residual during the investigated period, we find that it changes from 2.0 to 7.5 mm. Thus, the standard deviations can reach almost half of the amplitude of median range, which indicates the monthly averages cannot represent the variation during one month. In view of this, a higher temporal resolution gravity model can satisfy the need.

### 3.2. Time-Varying Annual Characteristics of Daily Vertical Displacements

**Figure 6:**
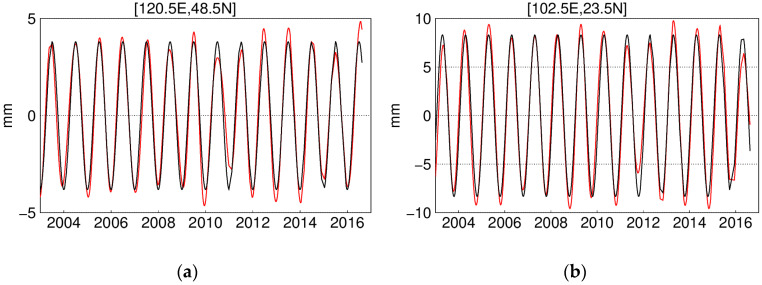

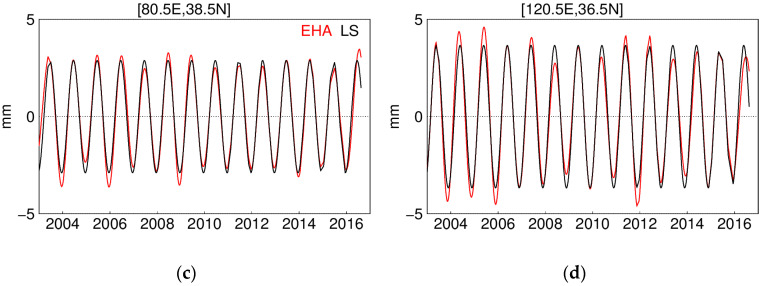
Annual signal extracted with enhanced harmonic analysis (EHA) and least square (LS) at selected grids: (**a**) at grid (120.5 E, 48.5 N); (**b**) at grid (102.5 E, 23.5 N); (**c**) at grid (80.5 E, 38.5 N); (**d**) at grid (120.5 E, 36.5 N).

**Figure 7:**
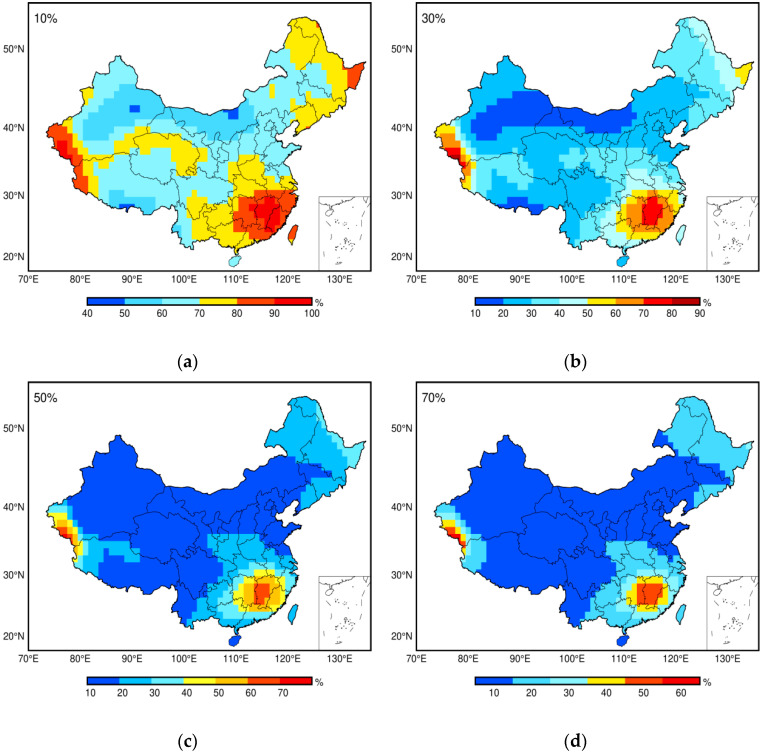
Percentage of epochs for annual signal bias related to LS: (**a**) larger than 10%; (**b**) larger than 30%; (**c**) larger than 50%; and (**d**) larger than 70%.

To extract the annual signal efficiently, EHA is applied to the GRACE-derived vertical displacement. From the example grids in Figure 6, we can see that the annual signal is indeed not in constant amplitude, and discrepancy is observed. Specifically, the relative annual signal bias is calculated as

(7) $$\mathrm{per}=\frac{\left|S_{\mathrm{EHA}}-S_{\mathrm{LS}}\right|}{\left|S_{\mathrm{LS}}\right|}\times 100\%$$

where $S_{\mathrm{EHA}}$ is the annual signal extracted with EHA, and $S_{\mathrm{LS}}$ represents the annual signal fitted with LS. The ranges of bias are sorted as larger than 10%, 30%, 50%, and 70%. The statistics shown in Figure 7 illustrate that the median percentage of epochs at grids with a bias larger than 10% is 69.7% and the proportions for the range of 30%, 50%, and 70% are about 30.1%, 18.4%, and 13.0%. This result indicates that the signal is obviously time-varying and the advantage of EHA over LS for obtaining such characteristics. From its spatial distribution, we can see that when analyzing the annual signal of Jiangxi province, the traditional LS with constant amplitude is impracticable. Moreover, we will go into the possible geophysical reasons that cause the time-varying signal in the future study.

## 4. Synthetic Time Series Analysis

To further validate the effectiveness of EHA application in time series, simulations are carried out in this section. First, to see whether EHA is able to extract the true signal with different types of noises, one single periodic signal with white noise, white and power-law noise, as well as white and Auto-Regression (AR) noise are separately generated. Further, to address whether EHA can distinguish the synthetic periodic signals in time series or not, series composed of two signals with time-varying amplitudes and white plus AR noise are simulated. Specifically, the time series are generated as Equations (3) and (4). Since the true signals are given, the root mean squared error (RMS) of the extracted and true signal is assessed. To keep consistent with the daily GRACE model in Section 3, the period spanning from January 2003 to August 2016 is selected. In total, there are 4992 days. In addition, the linear trend is assumed as 0.5 mm/yr, and the constant annual amplitude is 5.0 mm [27,28].

### 4.1. Impact of Noise in Different Types on Constant-Amplitude Signal

**Figure 8:**
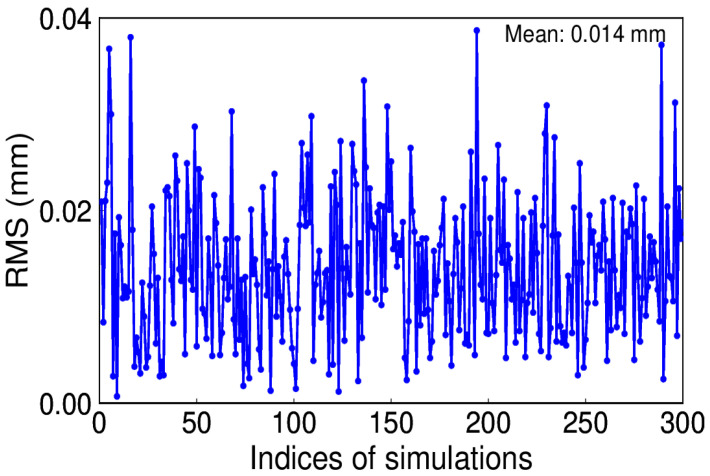
Root mean square (RMS) of extracted and simulated signal with white noise.

White noise is regarded as the commonly existing noise in time series. To be simple, assume white noise is the only noise and its amplitude is 0.8 mm. We conducted this simulation for 300 trials. Since a constant annual signal is given, the IP number is 1. Figure 8 depicts the RMS distribution, which reveals that the true signal can be effectively recovered by using EHA.

**Figure 9:**
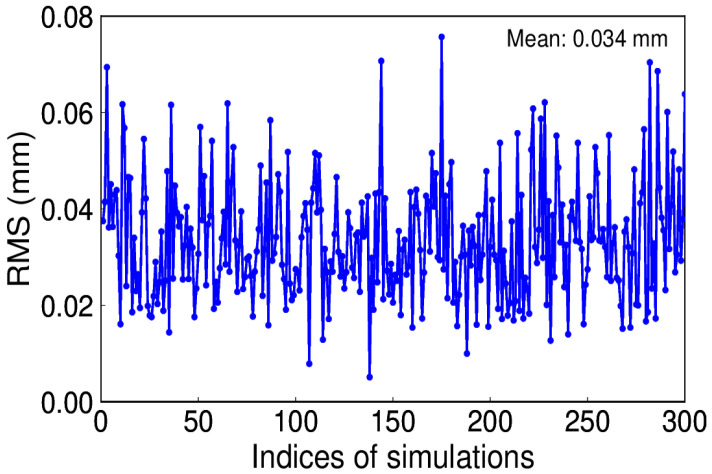
RMS of extracted and simulated signal with power-law noise.

Meanwhile, in many situations, not only white but also power-law noises are identified [29]. The amplitude of power-law noise is 0.8 mm/yr^−0.4^. Figure 9 shows the RMS of 300 simulations under white and power-law noise. Although it is larger than that of white noise, we can get the same conclusion in line with the white-only noise.

**Figure 10:**
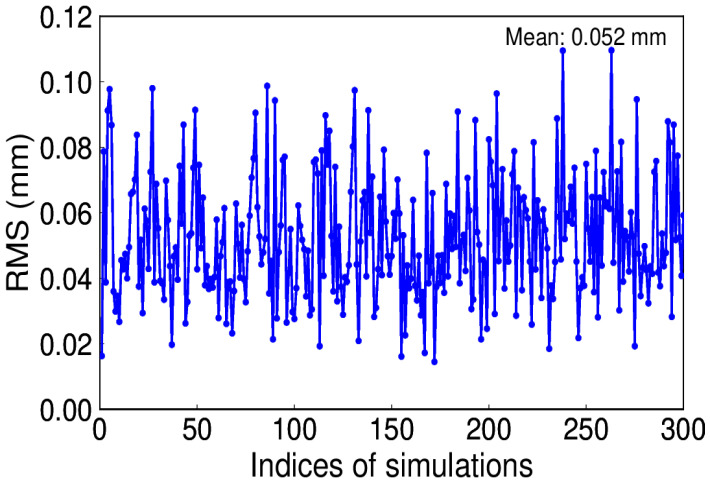
RMS of extracted and simulated signal with AR (3) noise.

For the daily ITSG-Grace 2018 model, the AR (3) model is optimal [23]. The simulated coefficients of AR (3) are 0.7, 0.5, and 0.3. The AR (3) noise amplitude is 0.8 mm. The RMS for the 300 simulations can be seen from Figure 10, which indicates that EHA can accurately extract the signal when AR (3) noise is present. However, from the comparison among the different noise, we can see that the existence of color noise could alias into the signal, which may affect the true signal extraction. Furthermore, the stochastic model of EHA, which still regards the color noise as white, is not appropriate.

### 4.2. Impact of Number of IPs on Time-Varying Annual Signal

**Figure 11:**
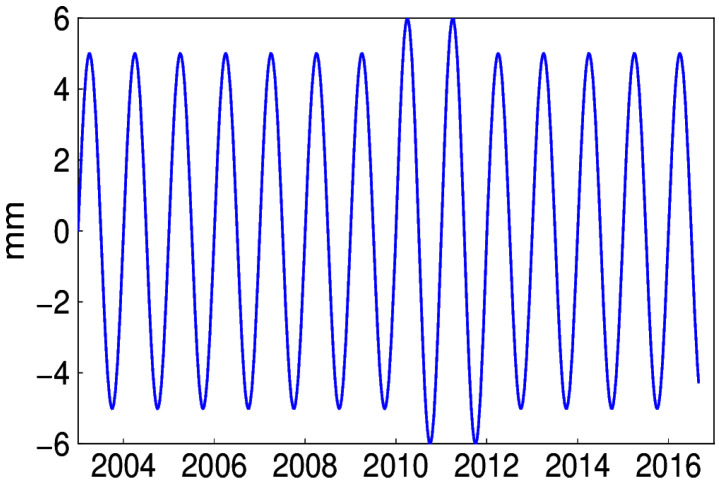
Time-varying annual signal simulated.

**Figure 12:**
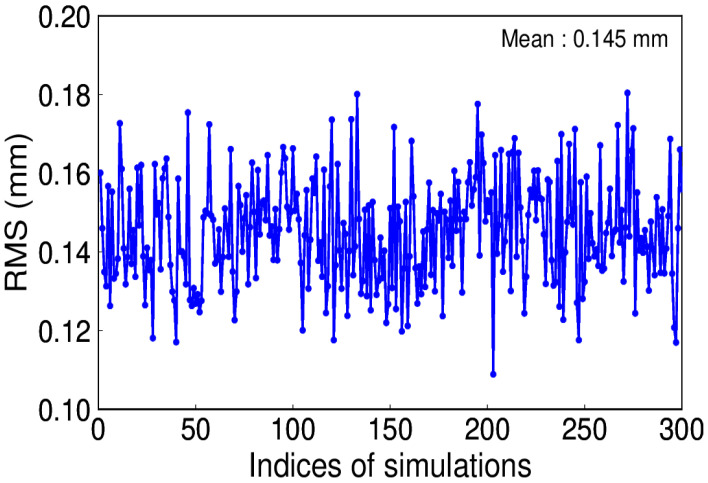
The minimum RMS with the optimal Independent Points (IPs).

**Figure 13:**
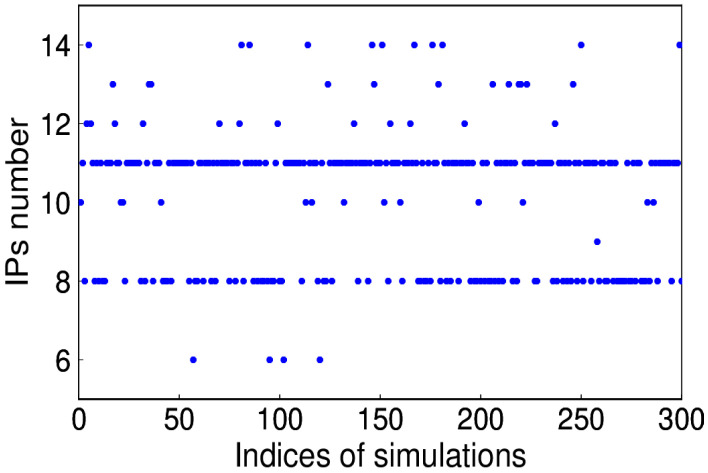
The optimal number of IPs determined on the minimum RMS criterion.

**Figure 14:**
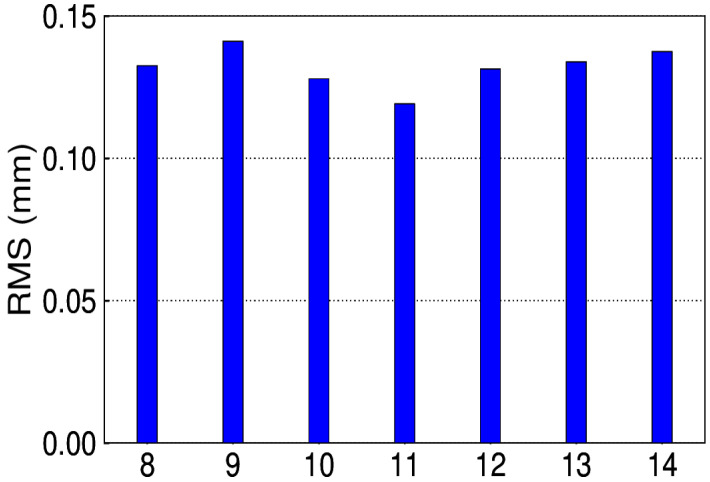
RMS for the IPs range from 8 to 14.

The simulated annual signal is shown in Figure 11, of which the amplitude is larger during the period from 2010 to 2011. Given the known signal in simulations, the number of IPs is determined on the criterion of minimum RMS of the extracted and simulated signal (see Figure 12). The corresponding IPs of the 300 simulations are presented in Figure 13. We can see that 8 and 11 account for a large proportion, that is, 30% and 52.3%, respectively. However, in fact, the above minimum RMS criterion to get the accurate IPs is not practicable, since the true signal is not known. Therefore, whether the bias of IPs causes substantial discrepancies is the question to address. Taking one simulation of which the optimal IPs is 11 for example, the IP ranges from 8 to 14 are tested. It is apparent that in Figure 14, the RMS differences exist, which is much lower than the amplitude of noise simulated.

### 4.3. Impact of Missing Data on Application of EHA

**Figure 15:**
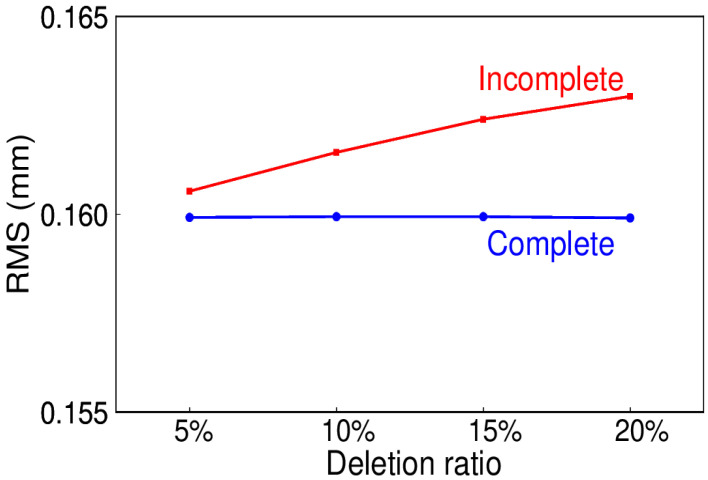
RMS for the signal extracted and the simulated.

Due to various reasons such as physical disturbance, equipment failure, or maintenance, missing data occasionally happen, which leads to the data being incomplete. In this test, randomly deletion of the simulations at the rate of 5%, 10%, 15%, and 20% are generated, and to get a comprehensive evaluation, 300 trials for each are carried out. The comparisons of complete and incomplete data are necessary. The mean RMS for the signal extracted and simulated is shown in Figure 15, the blue line represents the complete data, while the red one represents the incomplete data. It can be seen that as the data deletion ratio increases, the mean RMS increases. However, the relative percentage of mean RMS ranges from 0.4% to 1.9%, which could be neglected even as the deletion ratio reaches 20%. Meanwhile, for the daily ITSG-Grace2018 model, the days with insufficient observations are often regarded as the missing ones. The two longest continuous missing periods even last for two months, that is, from 1st August to 30th September 2013 and from 1st May to 30th June 2015, which is the representative experiment of the same missing data as the real data. The RMS for the complete and incomplete data is 0.163 and 0.176 mm, with the relative percentage of 7.9%. This confirms that EHA can deal with the missing data without interpolation beforehand, even for the continuously missing data.

### 4.4. Distinguishing Simultaneous Periodic Signals

**Figure 16:**
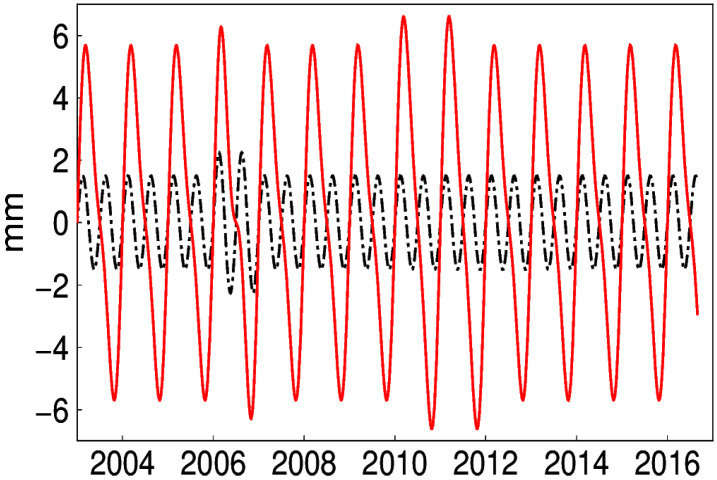
Time-varying semi-annual signal and synthetic signal.

**Figure 17:**
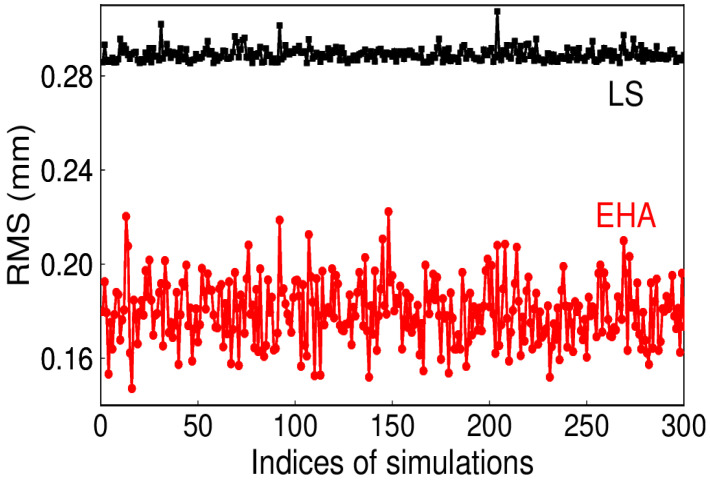
RMS for the signal extracted (by EHA and LS) and the simulated.

Actually, geodetic time series are synthesized with more than one harmonic signal, which is also evident in the frequency analysis [13]. The performance of EHA on obtaining a true signal from the simultaneous periodic signal is also tested. The time-varying semi-annual signals (in black) as well as its synthetic annual signal (in red) are shown in Figure 16. Since traditional LS cannot distinguish the time-varying signals, the RMS of LS is higher than that of EHA (see Figure 17). Therefore, EHA demonstrates its distinct advantage over LS. In addition, the mean RMS of 300 simulations for EHA is 0.18 mm, which indicates that the simultaneous signal extracted is close to the synthetic.

## 5. Discussion

Due to the temporal limit of the monthly gravity model, fast mass variation phenomena occurring on time scales shorter than month cannot be obtained. However, drought or flood usually do not last long. Meanwhile, in terms of inconsistent temporal resolution, both interpolation and averaging will lead to the loss of accuracy in signal extraction. For example, the displacements derived from the monthly gravity field are reduced from the daily GNSS coordinate time series to analyze the mass variation contribution on the crustal deformation. Thus, the development of a gravity model in higher temporal resolution is of great urgency. The ITSG-Grace 2018 is the first official released version of daily gravity model, and the applications highlight the advantage of higher temporal resolution [7]. Since GRACE is a powerful tool for monitoring mass loading, this study introduces ITSG-Grace 2018 in analyzing displacement. However, harmonics for the days with insufficient observations are regarded as unreliable. Therefore, missing data still occur in the daily gravity model. EHA can effectively deal with missing data and obtain the time-varying characteristics. The characteristics of vertical displacement are investigated, from which we can see that time-varying characteristics are very common. However, EHA has its own limits on the following aspects. First, it regards all the observations as having equal weight when solving the parameters, also without considering the existence of color noise. Second, although it has been confirmed that with a certain range, the effects on the signal extracted are slight, determining the number of IPs is subjective.

## 6. Conclusions

The vertical displacements in a 1.0° × 1.0° grid throughout China from January 2003 to August 2016 are derived from daily ITSG-Grace 2018. From the daily displacement time series, we can clearly see the sub-monthly variations. Meanwhile, the comparison of daily series and its monthly averages further reveal that daily resolution cannot be represented. Thus, a daily gravity model could be used to reflect the higher temporal resolution in applications such as the extreme flood, the contribution of mass loading on the daily GNSS coordinates, and so on. The periodogram indicates that the annual signal is the dominant signal, and EHA is applied to obtain it. The relative bias of the annual signal between EHA and LS is assessed. During the investigated period, we can find that the median percentage epochs for the grids with bias larger than 10%, 30%, 50%, and 70% is about 70%, 30%, 18%, and 13%, respectively. The statistics reveal that EHA can obtain time-varying characteristics, and from the spatial distribution, it is easy to find that time-varying cannot be ignored, especially in some specific regions.

The impact of different noise types, IP numbers, missing data, and simultaneous periodic signals are carried out to test the effectiveness of EHA. It can be concluded that no matter whether white or color noise exists, EHA can effectively extract the simulated signals. However, for the time-correlated noise, it could cause a spurious signal, which reduces the accuracy. Meanwhile, the stochastic model due to the existing of time-correlated noise should be introduced into EHA to improve its application in future study. Although the number of IPs affects the results of EHA, the findings in our simulations confirm that the fluctuation of IP numbers in a certain range will not bring the considerable impact. For dealing with the missing data, it is the most prominent feature of EHA that even for the continuously missing data, it can get the accurate signal. For the synthesized signal, EHA can deal with more than one periodic signal. From the comprehensive analysis of real data and simulations, given the simplicity of EHA and its adaptability to time series without any priori knowledge, we believe that EHA provides a promising option in analyzing the geodetic time series.
